# Neurofunctional Underpinnings of Audiovisual Emotion Processing in Teens with Autism Spectrum Disorders

**DOI:** 10.3389/fpsyt.2013.00048

**Published:** 2013-05-30

**Authors:** Krissy A.R. Doyle-Thomas, Jeremy Goldberg, Peter Szatmari, Geoffrey B.C. Hall

**Affiliations:** ^1^Department of Psychiatry and Behavioural Neurosciences, McMaster University, Hamilton, ON, Canada; ^2^Department of Psychology, Neuroscience and Behaviour, McMaster University, Hamilton, ON, Canada

**Keywords:** autism spectrum disorders, social cognition, audiovisual, emotion and functional magnetic resonance imaging

## Abstract

Despite successful performance on some audiovisual emotion tasks, hypoactivity has been observed in frontal and temporal integration cortices in individuals with autism spectrum disorders (ASD). Little is understood about the neurofunctional network underlying this ability in individuals with ASD. Research suggests that there may be processing biases in individuals with ASD, based on their ability to obtain meaningful information from the face and/or the voice. This functional magnetic resonance imaging study examined brain activity in teens with ASD (*n* = 18) and typically developing controls (*n* = 16) during audiovisual and unimodal emotion processing. Teens with ASD had a significantly lower accuracy when matching an emotional face to an emotion label. However, no differences in accuracy were observed between groups when matching an emotional voice or face-voice pair to an emotion label. In both groups brain activity during audiovisual emotion matching differed significantly from activity during unimodal emotion matching. Between-group analyses of audiovisual processing revealed significantly greater activation in teens with ASD in a parietofrontal network believed to be implicated in attention, goal-directed behaviors, and semantic processing. In contrast, controls showed greater activity in frontal and temporal association cortices during this task. These results suggest that in the absence of engaging integrative emotional networks during audiovisual emotion matching, teens with ASD may have recruited the parietofrontal network as an alternate compensatory system.

## Introduction

Broadly speaking, social-emotion perception relies heavily on the integration of multi-modal information, in particular audiovisual cues. A number of studies have examined audiovisual perception of social cues in autism spectrum disorders (ASD). Not all studies however agree on whether a behavioral impairment exists. Individuals with ASD have shown difficulty on tasks that require the matching of voice to face (Loveland et al., 1997; Boucher et al., 1998; Hall et al., 2003), the blending of audiovisual speech (de Magnee et al., 2008; Taylor et al., 2010), and lipreading (Smith and Bennetto, 2007). Conversely, other studies have reported no perceptual impairments in individuals with ASD when matching simple emotions in the face and voice (Loveland et al., 2008), assessing theory-of-mind using visual cartoons and prosody (Wang et al., 2006) and after being trained to integrate audiovisual speech cues (Williams et al., 2004). The discrepancy in findings may be due to differences in task complexity and among study samples in symptomatology, age, and cognitive ability.

Presently, neuroimaging studies have provided insight into brain activity in people with ASD and healthy controls during audiovisual emotion perception. To date, imaging studies have reported atypical activity in emotion and integrative regions in frontal and temporal lobes regardless of whether behavior was impaired (Hall et al., 2003), or preserved (Wang et al., 2006, 2007; Loveland et al., 2008). Some have reported hypoactivity in brain areas such as the inferior frontal cortex (Hall et al., 2003), medial prefrontal cortex (Wang et al., 2007), fronto-limbic areas (Loveland et al., 2008), superior temporal gyrus (Wang et al., 2007; Loveland et al., 2008), and fusiform gyrus (Hall et al., 2003) while other studies have reported increased activation of the inferior frontal cortex and temporal regions bilaterally when explicitly instructed to attend to certain social cues (Wang et al., 2006). This suggests that relative to controls, there are functional neurological differences underlying the way individuals with ASD process audiovisual emotion stimuli; and yet, despite these differences, it is possible for ASD individuals to perform successfully on audiovisual emotion tasks.

The compensatory neurofunctional activity observed in individuals with autism when dealing with multi-modal emotional cues is yet to be fully understood. Social cognition studies have shown that individuals with ASD do not demonstrate the preference for faces typically seen in controls when viewing social interactions (Volkmar et al., 2004). Moreover, there is evidence that people with ASD may shift their eye gaze away from the eye region of the face, limiting the depth of processing for the more salient emotional aspects of the face (Klin et al., 2002; Pelphrey et al., 2002; Dalton et al., 2005). By comparison, individuals with ASD have been found to be less impaired on auditory emotion processing (Kleinman et al., 2001), and may therefore favor the auditory domain over the visual domain (Macdonald et al., 1989; Sigman, 1993). Such observations raise the possibility that in ASD the perceptual challenges presented by audiovisual emotion stimuli may be met by changes in processing emphasis.

In the present functional magnetic resonance imaging (fMRI) study, we explored brain regions engaged during audiovisual emotion matching in ASD and examined (1) how brain activity differed from that observed during emotion matching in the visual and auditory modalities in isolation and (2) whether there are activation differences that distinguish individuals with ASD from controls during audiovisual emotion matching.

It has been suggested that the integration of audiovisual information is most beneficial when the signal in one modality is impoverished (Collignon et al., 2008). *Thresholding* the amount of visual emotion cues in the face is one way of limiting information in one modality. This technique has been used in the literature to study the onset of emotion perception in a number of special populations (Adolphs and Tranel, 2004; Graham et al., 2006; Heuer et al., 2010), to explore the developmental trajectory of sensitivities to emotional display (Thomas et al., 2007), and to examine the effects of various medications on improving emotion recognition (ER) (Alves-Neto et al., 2010; Marsh et al., 2010). In the present study we first established individual ER thresholds for facial stimuli which had reduced emotional intensities. Thresholded intensities were established for each participant on each emotion type in order to increase the processing advantage for integration and equate the behavioral performance across participants.

## Materials and Methods

Ethics approval for this study was obtained from St. Joseph’s Healthcare Research Ethics Board, Hamilton, ON, Canada. Participants who were 16 years old or older gave informed consent, while younger participants gave informed assent together with their parent’s consent. All participants were compensated for their time and travel expenses.

### Participants

Thirty-seven ASD and TD boys between the ages of 13 and 18 years (ASD = 21; TD = 16) participated in a series of pre-fMRI orientation and training procedures before undergoing an MRI scan. Teens with ASD were recruited from clinical and research programs for persons with ASD in Hamilton and Toronto, ON, Canada. Controls were recruited from local schools in the community. All teens with ASD carried a previous formal diagnosis of ASD, which was confirmed using the Autism Diagnostic Observation Schedule (ADOS; Lord et al., 2000) in 16 of the 18 ASD participants at the time of the scan. One teen in our ASD group fell short of the diagnostic cut-off for ASD by 1 point on the communication and reciprocal social interaction total score, and another teen was unable to stay or return for the ADOS testing because of his commute. Both these participants had clinically confirmed diagnoses of ASD by expert clinicians. ASD teens demonstrated good language abilities during the pre-fMRI orientation and training, and ADOS assessment. All participants had a non-verbal IQ (NVIQ) above 70 based on the Leiter International Performance Scale – Revised (Roid and Miller, 1997). None of the participants acknowledged a current or past history of substance abuse/dependence, or any major untreated medical illness. In addition, controls had no current or past neurological or psychiatric disorders, or a first-degree relative with ASD.

#### Final ASD group

A summary of our participant characteristics is presented in Table 1. Eighteen teens with ASD passed through all phases of training and participated in the final experiment (nine Asperger’s syndrome, five Pervasive Developmental Disorder – Not Otherwise Specified, three with the diagnosis of ASD and one with Autism). Eight of our ASD teens carried comorbid diagnoses (ADHD, Attention Deficit and Hyperactivity Disorder; CAPD, Central Auditory Processing Disorder; Visual Perceptual Learning Disorder; and Encopresis) and five of those carrying an ADHD diagnosis were on medication at the time of the scan. Sixteen ASD teens were right handed, as confirmed by the Edinborough Handedness Inventory (Oldfield, 1971).

**Table 1:** **Participant characteristics**.

Variables	TD controls Mean ± SD	ASD Mean ± SD	*t*	*p*
Number of participants	16	18		
Age	14.69 ± 1.70	14.94 ± 1.55	0.46	0.65
NVIQ	117.94 ± 12.73	97.72 ± 13.52	4.47	0.05^*^
Handedness	*R* (*n* = 16)	*R* (*n* = 16), *L* (*n* = 2)		
ADOS
Communication		*n* = 17, 5.67 ± 3.06		
Social		*n* = 17, 9.20 ± 3.03		
Emotion pre-test scores
Face emotion	14.75 ± 1.06	13.89 ± 1.78	1.68	0.10
Voice emotion	11.56 ± 1.86	10.78 ± 1.83	1.24	0.23
Emotion threshold
Happy	23.44 ± 3.52	27.22 ± 5.48	2.36	0.02^*^
Sad	44.06 ± 14.05	40.56 ± 15.71	0.68	0.05
Angry	47.19 ± 16.12	50.56 ± 13.05	0.67	0.51

Demographic information and baseline scores for the TD and ASD groups are noted in means and standard deviations, with *t*- and *p*-values indicated. Significant group differences are indicated by the use of an asterisk*. Terms such as non-verbal IQ (NVIQ), Autism Diagnostic Observational Schedule (ADOS), and standard deviation (SD) were abbreviated to their acronyms.

#### Final typically developing control group

Sixteen TD boys were group matched with the ASD group on chronological age (see Table 1). All TD controls were right handed, as confirmed by the Edinborough Handedness Inventory (Oldfield, 1971).

### Stimuli

**Figure 1:**
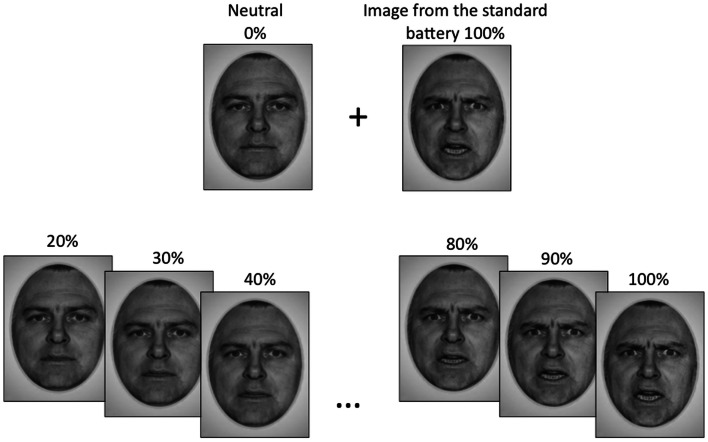
**The emotional face stimuli used in this study were generated by morphing a neutral face with an emotional image of the same actor from the standard face battery, to obtain gradations of that emotion**. Gradations began at 20% emotion content and increased in increments of 5% up to a maximum of 100%, which was the standard image.

Standardized photographs of faces expressing the emotions of happiness, sadness, and anger (Ekman and Friesen, 1976 and NimStim^1^) were morphed with pictures of neutral expressions from the same actor to create a battery of graded emotion face stimuli (Abrosoft FantaMorph software^2^). The graded emotion stimulus set began at 20% emotion intensity content, and were incremented in intervals of 5%, up to and including 100% emotion content for each face. Thus, there were 17 facial images, plus a neutral image, for each individual face. Fifteen faces were used for each emotional expression (eight female and seven male for happy and sad, seven female and eight male for angry), to generate a total of 810 face stimuli (15 faces × 18 facial images × 3 emotions). Examples of our graded emotional faces are shown in Figure 1.

The auditory stimuli were .wav files made from recordings of male and female actors reciting a series of semantically neutral phrases (for example: “where are you going;” “what do you mean;” “I’m leaving now”) with neutral or emotionally prosodic emphasis (happy, sad, or angry). A total of 103 clips were equalized to a preset maximal volume, and set at a maximum duration of 2.8 s. The prosodic stimuli were validated in a group of six healthy young adults, with auditory recordings that received the highest agreement of emotion type (88.4% or greater inter-rater agreement) and strongest intensity ratings (80% or greater inter-rater agreement) used as experimental stimuli (*n* = 56).

### Pre-fMRI emotion recognition test

Prior to scanning, a behavioral pre-test was conducted to ensure each participant could identify the emotions used in the study, and to assess whether the two groups were performing the ER task at a comparable level. In this computerized task, teens viewed 16 emotion faces and heard 16 emotion voices (4 for each emotion type), which were different from the set used in the fMRI paradigm. Stimuli were presented with the four possible emotion labels (happy, sad, angry, and no emotion) and teens were asked to choose the emotion label that best described the face or voice.

### Emotion recognition threshold

Participant-specific emotional recognition thresholds for each emotion type were established prior to scanning. In a computerized behavioral test, each teen was presented with a matching task in which an emotional face and label appeared on the screen. The teen was asked to decide if the emotion in the face was a “match” or a “mismatch” to the emotion label. This pre-test used a face battery with stimuli (happy, sad, angry, and neutral) that were distinct from those used in the fMRI paradigm. Emotion types were randomly presented. The initial emotional intensity of the faces in this task was set at 70% and then was adaptively reduced in increments of 5%, when the participant correctly identified an emotion at each threshold four times. The intensity level (%) at which the teen failed four trials out of eight successive presentations of an emotion was set as the teen’s specific “threshold” for that emotion. Full valance emotional faces (100%) and emotional faces at each teens personal threshold were used later in the fMRI tasks.

### Imaging tasks

**Figure 2:**
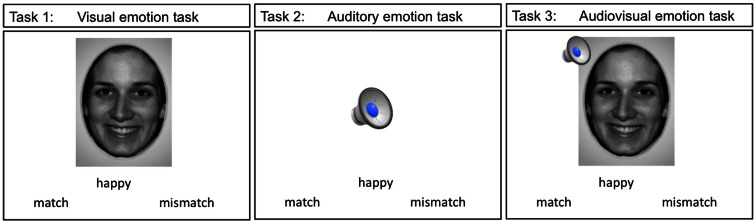
**The three imaging emotion tasks used in this study are depicted**. Stimuli in tasks 1 though 3 were presented with an emotion label and teens were asked to indicate whether the stimuli were a “match” or a “mismatch” to the label displayed.

A total of three event-related paradigms were used in the present study. These three tasks are shown in Figure 2. Teens were presented with an emotion label and either a static emotion face (visual emotion), a spoken emotion sentence (auditory emotion), or both face and voice stimuli simultaneously (audiovisual emotion), with a forced choice option of “match” or “mismatch.” Teens used MRI compatible response buttons to identify whether the emotion stimulus matched the displayed emotion label. The words “match” and “mismatch” appeared to the right and left of the center of the screen. When the teen made a selection the font color changed from black to blue to highlight the selection. The face stimuli in all the tasks consisted of 4 emotion faces at the full emotion level (100%) and 9 emotion faces at the teen’s thresholded level, for a total of 13 trials per emotion type. Each task had a total of 52 trials (13 trials × 4 emotion types).

### fMRI data acquisition

In the scanner, visual stimuli were projected onto a visor that sat on top of the head coil (MRIx systems, Chicago, IL, USA) and auditory stimuli were presented using MRI compatible sound isolation headphones (MR Confon, Germany). Responses were made via a hand-held response pad. Stimulus presentation was done using E-PRIME software (Psychology Software Tools, Pittsburgh, USA) and errors were collected across all 3 paradigms. Participants were scanned using a GE Signa 3T scanner equipped with an 8 parallel receiver channel head coil. A routine 3D SPGR scan for detailed anatomy was acquired prior to functional scanning (3D SPGR pulse, sagittal plane, fast IRP sequence, TR = 10.8 ms, TE = 2 ms, TI = 400 ms, flip angle = 20 °, matrix 256 × 256, FOV = 24, slice thickness 1 mm, no skip). For the single modality paradigms the functional images were acquired with a gradient-echo planar imaging (EPI) sequence, with 36 axial contiguous slices (3 mm thick, no skip) encompassing the entire cerebrum [repetition time/echo time (TR/TE) 3000/35 ms, flip angle = 90°, field of view (FOV) 24 cm, matrix 64 × 64]. For the crossmodal paradigm, fMRI images were acquired with the same scan parameters as above but with a TR of 2500 to provide sufficient time for stimuli presentation and perceptual processing (each stimulus was presented for two TRs).

All three paradigms were presented as event-related designs. Emotion trials were presented with variable jittered interstimulus intervals (range: 2.5–12.5 s) during which time a fixation screen was presented. The total scan time for the unimodal tasks was 7 min 24 s and for the crossmodal task, 8 min 30 s, with a total scan time of 35 min (approximately 25 min total task time + anatomical and LOC scan).

### Data analysis

Functional data was processed using BrainVoyager QX version 2.0.7 (Brain Innovation B.V., Maastricht, Netherlands) to identify regions of activation during each task. The functional data was co-registered to the seventh image in the series to correct for any subtle head motion during the functional run. Volumes that showed transient head motion beyond 2 mm in any direction were removed from the series. This resulted in the deletion of 610/11,772 volumes in the ASD group and 151/10,464 volumes in the control group. Realigned images were spatially normalized into standard stereotactic space. These images were smoothed with a 6 mm full-width half maximum Gaussian filter to increase signal to noise ratio and to account for residual differences in gyral anatomy. Activation maps were constructed identifying clusters of activity associated with the peak differences in activation both within group and between groups. Group differences were identified through a second-level random effects model to account for inter-group variability.

Behavioral statistical analysis was carried out using paired (for within group) and unpaired (for between group) *t*-tests in SPSS (2009, Chicago, IL, USA) with the threshold for significance set at *p* < 0.05.

## Results

### Sample overview

Participant baseline scores are summarized in Table 1. On the pre-fMRI ER test, ASD participants and controls did not differ on their ability to identify the emotion in 16 faces (*p* = 0.10) and 16 voices (*p* = 0.23) (see Table 1 for complete details). Non-verbal intellectual functioning was in the normal range for both groups. However, the ASD group had a lower estimated NVIQ than the controls (*p* < 0.05) (see Table 1 for complete details). Pearson correlation analysis was conducted to examine the relationship between NVIQ and pre-fMRI ER scores in each test group. No significant correlation between NVIQ scores and face, and voice emotion pre-test scores were found for either group (ASD face ER and IQ correlation: *r* = 0.23, *p* = 0.37; ASD voice ER and IQ correlation: *r* = 0.29, *p* = 0.24; TD face ER and IQ correlation: *r* = 0.03, *p* = 0.92; TD voice ER and IQ correlation: *r* = 0.17, *p* = 0.54).

On the ER threshold test, no significant group differences were found for sad thresholds (*p* = 0.50) and angry thresholds (*p* = 0.51) (see Table 1 for complete details). However, teens with ASD had a significantly higher threshold for happy, compared to controls (*p* < 0.05) (see Table 1 for complete details).

### Behavioral results during fMRI

Participant accuracy during the three fMRI tasks are summarized in Table 2. On the *visual emotion task* teens with ASD, compared to controls made significantly more errors in matching emotional faces to an emotion label (*p* < 0.05) (see Table 2 for complete details). However, there were no accuracy differences between groups on the *auditory emotion task* (*p* = 0.07) or the *audiovisual emotion task* (*p* = 0.11) (see Table 2 for complete details).

**Table 2:** **Accuracy scores on emotion matching tasks**.

	ASD Mean accuracy ± SD (%)	TD Mean accuracy ± SD (%)	*t*	*p*
Visual emotion task	70.11 ± 6.69	76.75 ± 5.42	3.15	*p* < 0.05^*^
Auditory emotion task	79.06 ± 6.86	83.25 ± 6.13	1.87	*p* = 0.07
Audiovisual emotion task	77.44 ± 7.11	81.56 ± 7.55	1.64	*p* = 0.11

Accuracy scores for the ASD and TD groups on emotion matching tasks are noted in means and standard deviations, with *t*- and *p*-values indicated for each emotion task. Significant group differences are indicated by the use of an asterisk ^*^.

### Functional activation results

#### ASD – within group results

##### Audiovisual emotion matching compared to visual emotion matching

Complete details pertaining to activation differences are presented in Table 3A. Individuals with ASD activated frontal and temporal regions during both audiovisual and visual emotion matching, although more frontal regions were activated during visual processing. Audiovisual processing also engaged the cuneus (BA 19). In comparison, visual emotion matching recruited regions in the limbic cortex (BA 23) and the basal ganglia (caudate and thalamus).

**Table 3:** **Significant brain activation differences observed between audiovisual, and visual or auditory emotion matching in teens with and without ASD**.

(A) TEENS WITH ASD		
**Audiovisual compared to visual emotion matching**		
	Audiovisual > visual	Visual > audiovisual

Frontal	BA 8 (−28, 22, 48) *t* = 4.40	BA 10 (23, 58, 12) *t* = 5.00, BA 6 (44, −2, 42) *t* = 6.84, BA 24 (11, 16, 27) *t* = 4.08
Parietal	BA 19 (−32, −92, 33) *t* = 4.60 (cuneus)	
Temporal	BA 19 (−49, −83, 21) *t* = 4.45, BA 22 (−68, −38, 9) *t* = 4.63	BA 22 (44, −53, 15) *t* = 4.11, BA 30 (20, −35, 3) *t* = 5.85
Occipital		BA 19 (35, −71, -9) *t* = 9.75 (FFA)
Limbic		BA 23 (−4, −20, 27) *t* = 5.85
Subcortical		Caudate (17, −35, 18) *t* = 4.15, Thalamus (2, −32, 3) *t* = 4.75, (−19, −35, 3) *t* = 5.84
**Audiovisual compared to auditory emotion matching**		

	Audiovisual > auditory	Auditory > audiovisual

Frontal	BA 11 (−22, 55, −12) *t* = 2.98, (−40, 46, −13) *t* = 3.80, (−7, 40, −15) *t* = 3.20, BA 10 (−4, 52, 9) *t* = 4.23, BA 6 (−25, 13, 60) *t* = 4.77	BA 10 (18, 67, 21) *t* = 3.20, BA 47 (−16, 7, −18) *t* = 3.13
Parietal	BA 2 (35, −29, 39) *t* = 5.21, BA 39 (−43, −71, 27) *t* = 3.34, (47, −77, 24) *t* = 3.35	
Temporal	BA 20 (−52, −32, −18) *t* = 3.91	BA 41 (−52, −26, 6) *t* = 10.22, BA 38 (35, 10, −24) *t* = 3.51, BA 20 (−43, −17, −30) *t* = 4.27
Occipital	BA 19 (−32, −89, 39) *t* = 3.17, BA 18 (−1, −102, 15) *t* = 4.96, (14, −95, 18) *t* = 3.34	
Limbic		Amygdala (17, −8, −9) *t* = 3.15
**(B) CONTROLS**		
**Audiovisual compared to visual emotion matching**		

	Audiovisual > Visual	Visual > Audiovisual

Frontal	BA 10 (−33, 71, 6) *t* = 4.76, (−43, 61, 9) *t* = 4.81	BA 9 (−37, 40, 27) *t* = 5.41, BA 8 (29, 46, 39) *t* = 4.85, (29, 37, 57) *t* = 4.17, BA 6 (47, −2, 33) *t* = 6.28
Parietal		BA 40 (41, −32, 30) *t* = 4.20
Temporal	BA 42 (−68, −26, 9) *t* = 5.55, BA 21 (69, −14, −3) *t* = 5.66, (−58, −2, −6) *t* = 3.89, BA 37 (−61, −59, −6) *t* = 5.43	BA 13 (−40, −14, −21) *t* = 5.45, BA 20 (32, −14, −27) *t* = 5.42
Occipital	BA 19 (−61, −74, 18) *t* = 4.21	
Limbic		Hippocampus (−31, −14, −24) *t* = 4.15
**Audiovisual compared to auditory emotion matching**		

	Audiovisual > auditory	Auditory > audiovisual

Frontal	BA 10 (−37, 46, 3) *t* = 4.18, BA 11 (17, 40, −15) *t* = 4.48, BA 6 (23, 19, 51) *t* = 4.68, (−22, 10, 48) *t* = 5.57	
Parietal	BA 31 (−4, −47, 33) *t* = 3.79	BA 7 (−22, −68, 60) *t* = 4.65
Temporal	BA 37 (−31, −41, −9) *t* = 3.91	BA 41 (53, −20, 3) *t* = 14.84, BA 20 (41, −8, −30) *t* = 3.98
Occipital	BA 19 (44, −86, 24) *t* = 3.85, (32, −44, −3) *t* = 4.63, (−37, −80, 30) *t* = 6.07, BA 18 (5, −98, −3) *t* = 6.86	

*Significant differences in brain activity during audiovisual emotion matching and visual and auditory emotion matching in teens with ASD (A) and controls (B). Brodmann areas (BA) are presented and coordinates are given in Talairach space (medial-lateral – *x*, anterior-posterior – *y*, and superior-inferior – *z*). Positive values are right, anterior and superior. All *t*-values are above the threshold for significance (corrected for multiple comparisons using the False Discovery Rate, *p* = 0.05)*.

##### Audiovisual emotion matching compared to auditory emotion matching

Complete details are outlined in Table 3A. Both audiovisual and auditory emotion matching engaged frontal and temporal regions. However, more frontal areas were recruited during audiovisual processing, while more temporal areas and the amygdala showed greater activation during auditory processing. Audiovisual emotion matching also engaged parietal regions such as the postcentral gyrus (BA 2) and the angular gyrus (BA 39).

#### Controls – within group results

##### Audiovisual emotion matching compared to visual emotion matching

Complete activation details are presented in Table 3B. In typically developing teens, audiovisual, and visual emotion matching engaged frontal and temporal brain regions, although more frontal areas were recruited during visual processing and more temporal areas during audiovisual processing. Audiovisual processing also engaged occipital regions such as BA 19. Visual emotion matching additional recruited the inferior parietal lobule (BA 40), and the hippocampus.

##### Audiovisual emotion matching compared to auditory emotion matching

Full activation details are shown in Table 3B. Both audiovisual and auditory emotion matching activated temporal and parietal regions. Audiovisual emotion processing additionally recruited frontal and occipital brain areas.

#### Between groups – Audiovisual emotion matching

**Figure 3:**
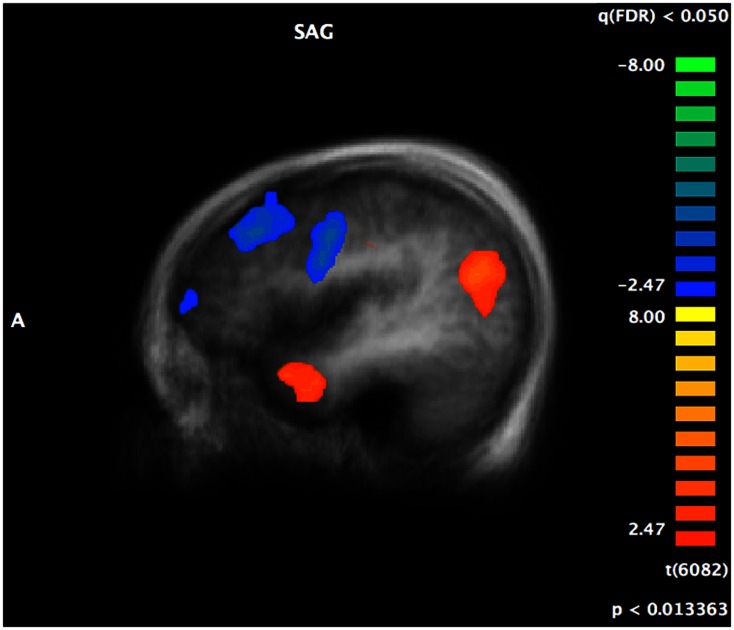
**A t-map showing regions of the brain activated more in controls (in blue) compared to regions activated more in individuals with ASD (in red) during the crossmodal emotion matching**. The activation differences are visible from *x* = −43 (left hemisphere).

Complete details of group activation differences are noted in Table 4 and group differences visible from *x* = −43 are shown in Figure 3. A number of frontal and temporal regions were activated in both the ASD and control groups. However, more frontal and temporal activation was observed in controls. Participants with ASD activated parietal regions, namely BA 39 and 7 more than controls, while controls activated BA 18 in the occipital lobe and the hippocampus more.

**Table 4:** **Significant differences in brain activity in teens with and with ASD during audiovisual emotion matching**.

	ASD > controls	Controls > ASD
**AUDIOVISUAL EMOTION MATCHING**		
Frontal	BA 9 (−1, 49, 27) *t* = 5.60, BA 46 (−37, 31, 21) *t* = 3.23, BA 11 (−28, 37, −15) *t* = 3.57, BA 8 (23, 28, 36) *t* = 3.92	BA 10 (15, 64, −9) *t* = 3.74, (−40, 52, 9) *t* = 3.08, (−31, 62, 12) *t* = 2.89, (32, 58, 22) *t* = 3.85, BA 46 (−52, 41, 24) *t* = 3.55, BA 6 (2, 1, 51) *t* = 3.57, BA 47 (23, 19, −12) *t* = 3.15, (41, 22, −12) *t* = 3.82, BA 32 (8, 28, 24) *t* = 3.34, BA 13 (41, 4, 18) *t* = 5.42, BA 9 (−43, 22, 36) *t* = 4.68, BA 6 (−34, −8, 30) *t* = 5.34, BA 23 (−1, −35, 20) *t* = 4.14, (8, −17, 28) *t* = 3.46
Parietal	BA 39 (−46, −68, 18) *t* = 5.36, BA 7 (−10, −50, 36) *t* = 6.97	
Temporal	BA 21 (−67, −32, −6) *t* = 4.95, (53, −5, −21) *t* = 3.99, BA 30 (29, 16, 24) *t* = 3.23	BA 22 (50, −20, −6) *t* = 3.62, BA 21 (−61, 2 −9) *t* = 3.10, BA 30 (−19, −38, 6) *t* = 4.51, BA 36 (38, −26, −18) *t* = 3.51
Occipital		BA 18 (−37, −87, −12) *t* = 10.69
Other		Hippocampus (35, −8, −15) *t* = 2.88

*Significant differences in brain activity between teens with ASD and controls during audiovisual emotion matching. Brodmann areas (BA) are presented and coordinates are given in Talairach space (medial-lateral – *x*, anterior-posterior – *y*, and superior-inferior – *z*). Positive values are right, anterior and superior. All *t*-values are above the threshold for significance (corrected for multiple comparisons using the False Discovery Rate, *p* = 0.05)*.

## Discussion

The findings of the present study suggest that individuals with ASD use integrative cortices when processing audiovisual emotion stimuli, however these cortices were different than the typical integration network observed in typically developing controls. During audiovisual emotion matching teens with ASD showed greater engagement than controls in the parietofrontal network; circuitry suggested to be involved in attention modulation and language processing (Silk et al., 2005). Conversely, controls showed more typical activation of established functional networks associated with integration and emotion processing in frontal and temporal regions of the brain (Hall et al., 2003; Wang et al., 2006, 2007; Loveland et al., 2008). These findings may suggest a compensatory network that individuals with ASD relied on when processing audiovisual emotion stimuli.

The term “network” has been used in other research to refer to a group of brain regions commonly activated during specific behaviors, including social cognition, attention, integration, and language (Mesulam, 1990; Sowell et al., 2003; Baron-Cohen and Belmonte, 2005; Silk et al., 2005). The parietofrontal “action-attentional” network (Silk et al., 2005) consists of a group of frontal (BA 46, 10, and 8) and parietal (BA 39, 40, and 7) brain areas involved in modulating one’s attention in preparation to react to a stimulus (see Cohen, 2009 for a review). Activity in this network and particularly in BA 39/40 is important for both auditory and visual goal-directed behavior (see Cohen, 2009 for a review). Indeed the between group analysis showed significantly greater activity in teens with ASD, compared to controls in a similar network (frontal: BA 46, 9, 8, and parietal: 39, 7). These findings may suggest that teens with ASD relied on this network for attentional and integrative purposes.

In addition, studies show that BA 39 (Hoenig and Scheef, 2009; Monti et al., 2009) and nearby “supporting” areas; BA 7 and 40 (Monti et al., 2009) are activated when typically developed individuals draw linguistic/semantic inferences. In a similar vein, it has been suggested that BA 39 in concert with the precuneus, the superior parietal lobule, and the middle frontal gyrus are implicated in understanding language cues in context (Martín-Loeches et al., 2008). Thus it is possible that during audiovisual emotion matching teens with ASD may have relied more heavily on cues in the auditory stimuli than features in both the auditory and visual domains.

There are some limitations in the present study. It may have been helpful to include a debriefing questionnaire that examined participant task strategy. However, this data was not collected. Secondly, given the limited number of teens with ASD available for enrollment, we were limited in our recruitment of teens who were medication free. As such, we included five participants who were on medication at the time of the scan. These medications included stimulants (Strattera and Biphentin), antipsychotic (Risperdal and Seroquel), anticonvulsant (Trileptal) and other medications used to treat side effects (namely Clonidine and Cogentin). As such we cannot rule out possible pharmacology influences on the observed brain activity in the ASD teens. Future studies should attempt to explore audiovisual emotion processing in unmedicated participants with ASD to confirm the current results. Lastly, our two groups also differed in intellectual capacity. We did find that our ASD group tested in the high functioning range and that their performances were similar in many ways to the controls. However, we cannot rule out the possibility that different response strategies were adopted by teens with ASD as a function of their cognitive abilities. Further work will be required to adequately address this concern.

In summary, the current study examined differences between the brain networks involved in audiovisual and single modality emotion matching in teens with and without ASD, and networks involved in audiovisual processing in teens with ASD compared to controls. Of note in teens with ASD, audiovisual emotion matching compared to single modality emotion matching elicited significantly greater activity in the parietofrontal network involved in attention modulation, goal-directed behavior and language comprehension. This activity was observed to be significantly greater in teens with ASD compared to controls during audiovisual emotion matching. In comparison, controls showed greater activity in frontal and temporal association areas during the audiovisual emotion task. These results suggest that in the absence of engaging integrative emotional networks during audiovisual emotion matching, teens with ASD may have recruited the parietofrontal network as an alternate compensatory system.

## Conflict of Interest Statement

The authors declare that the research was conducted in the absence of any commercial or financial relationships that could be construed as a potential conflict of interest.

## Footnotes

^1^http://www.macbrain.org/resources.htm

^2^http://www.fantamorph.com/
